# 측정도구의 심리계량적 속성 2: 구조타당도, 내적일관성 및 교차문화타당도/측정동일성

**DOI:** 10.4069/kjwhn.2021.05.18

**Published:** 2021-06-17

**Authors:** Eun-Hyun Lee

**Affiliations:** Graduate School of Public Health, Ajou University, Suwon, Korea; 아주대학교 보건대학원

**Keywords:** Cultural validity, Internal consistency, Instrument, Psychometrics, Structural validity, 문화타당도, 측정도구, 내적일괄성, 심리계량적 속성, 구조타당도

## Abstract

Structural validity, internal consistency, and cross-cultural validity/measurement invariance are psychometric properties of the internal structure of an instrument. In psychometric studies published in Korean nursing journals, structural validity has mainly been assessed using confirmatory factor analysis. Cross-cultural validity/measurement invariance has rarely been evaluated. It is recommended for Korean nursing researchers to evaluate the internal structure of instruments using a greater variety of methods, such as item response theory, Rasch analysis, multi-group confirmatory factor analysis, and differential item functioning.

## 서론

측정도구 개발과정에서 내용타당도 다음으로 실시되어야 할 것은 도출된 예비문항들이 어떻게 척도(하부척도)로 결합되는지를 결정하는 일이다. 다시 말해서, 측정도구의 내적구조(internal structure)를 결정하는 것이다. 심리계량적 속성(psychometric properties) 중에서 도구의 내적구조를 파악하기 위해 실시되는 것은 구조타당도(structural validity), 내적일관성(internal consistency) 및 교차문화타당도/측정동일성(cross-cultural validity/measurement invariance)이다[1].

## 구조타당도

구조타당도는 개발하려는 도구가 측정하고자 하는 구성개념의 차원(dimension)을 적절히 반영하는가에 관한 것으로, 주로 사용되는 검증방법에는 확인적 요인분석(confirmatory factor analysis, CFA)과 문항반응이론(item response theory, IRT)/라쉬 분석(Rasch analysis)이 있다[2]. 여기서는 통상적으로 많이 사용되는 CFA에 대해 살펴볼 것이다.

측정도구의 구조타당도 평가를 위한 CFA 사용은 이론 또는 실증적 근거에 입각해서 잠재변수(요인)와 관찰변수(문항)의 관계 및 잠재변수들의 관계에 대해 가설적 측정모델을 만들고, 실제로 수집한 자료와 얼마나 일치하는지를 검증하기 위함이다. 측정모델은 차원에 따라 단일차원(unidimensional)과 다차원(multidimensional) 모델로 구분될 수 있다. 다차원 모델은 일차(first-order)와 고차(higher-order) 수준의 모델로 나눌 수 있고, 이외에도 이중요인(bi-factor) 모델이 있다. 모델의 선택은 구성개념에 대한 이론 및 실증적 근거에 의해 정해진다. 따라서 연구자는 CFA를 적용하기 전에, 관심 있는 구성개념의 이론적 배경이나 실증적 근거에 대해 충분히 파악하는 것이 중요하다.

만약 도구개발에서 측정하고자 하는 구성개념과 관련된 어떤 이론이 사용되었다면, 이를 바탕으로 이미 내용타당도 단계에서 개념적 차원과 각 차원에 속하는 문항들이 정해진 상태일 것이다. 따라서 가설로 세워진 측정모델에 대해 CFA를 실시한다. 하지만 어떤 경우에는 내용타당도에서 구성개념과 관련된 문항들을 도출하였지만, 개념적 차원의 수를 모르거나 도출된 문항들이 어떤 차원에 어떻게 군집되는지에 대한 정보가 없을 수 있다. 이런 경우에는 먼저 탐색적 요인분석(exploratory factor analysis, EFA)을 실시하여 요인의 수와 어떤 문항이 어떤 특정 요인에 의미 있게 연관이 있는지를 확인한다. 이렇게 확보된 실증적 근거를 바탕으로 CFA를 실시한다[3].

사례로 Lee 등[4]이 개발한 당뇨병 환자의 헬스 리터러시 측정도구(diabetes health literacy scale, DHLS)를 살펴보자. 최근 의료보건 분야에서 헬스 리터러시 개념의 중요성이 부각되고 있지만, 상대적으로 이에 대한 이론은 초기단계에 있다. 따라서 측정모형에 사용할 이론적 기틀을 찾기 힘든 상황이라고 할 수 있다. 연구자들은 구성개념이 몇 개의 요인으로 이루어지며 어떤 개념적 속성들이 어떤 요인에 속하는지에 대한 실증적 근거를 만들고, 이 근거를 기틀로 측정모형을 구성해서 CFA를 수행하였다. 즉, 462명의 당뇨병 환자를 모집하고 이를 무작위로 두 개의 표본으로 나누어서, 첫 번째 표본에는 EFA를 실시하여 3개의 요인으로 구성되었다는 것과 각 문항들이 어떤 요인에 속하게 되는지에 대한 실증적 정보를 얻었다. 이렇게 얻은 실증적 정보를 바탕으로, 두 번째 표본으로 CFA를 실시하여 DHLS 구조타당도를 검증하였다. 즉, 각각의 표본에 EFA와 CFA를 적용하여 구조타당도를 교차확인(cross-validation)하는 방법을 사용하였다.

CFA를 수행하기 위해 고려해야 할 사항 중에 하나는 표본크기이다. 일반적으로 많이 사용되는 기준(rule of thumb)은 추정되는 모수의 10배 이상이며, 최소 200 케이스가 사용된다[3]. 예를 들어 측정모형에 두 개의 요인이 있는데, 첫 번째 요인에는 7개의 문항이 있고 두 번째 요인에는 2개의 문항이 있으며 두 개의 요인은 상호관련이 있다고 하자(first-order two-factor model with a total of nine items). 이 측정모델에서 추정되어야 할 모수는 19로, 필요한 표본크기는 최소 190 (19×10)이 된다. 하지만 최소 200케이스 기준을 고려한다면, 200 이상이 필요하다. 표본크기는 이 외에도 자료의 정규성, 요인의 개수 및 복잡성 등을 고려해서 정해야 한다.

측정모델에 대한 가설이 만들어졌으면, 사용할 통계 프로그램을 결정하고 수집된 자료를 가지고 CFA를 실시한다. CFA을 위한 모수 추정 방법으로 가장 흔히 사용되는 것은 최대우도법(maximum likelihood)으로 대부분의 프로그램에서 초기 설정되어 있다[5]. 최대우도법을 사용하기 위해서는 다변량 정규성(multivariate normality) 가정을 만족해야 한다. 하지만 문항의 점수가 Likert 척도 유형(예를 들어 1, 매우 동의함; 2, 동의함; 3, 동의하지 않음; 4, 전혀 동의하지 않음)의 서열 척도이면, 특히 문항반응의 범주수준의 개수가 2–4일 경우 다변량 정규성을 만족하기 어려워서 편중된 모수 추정이 이루어진다. 다변량 정규성은 Mardia’s normalized estimate로 검증할 수 있다[6]. 검증 결과 다변량 정규성의 가정이 위배되면, 다른 추정방법을 고려하거나(예를 들어 weighted least squares, asymptotically distribution-free, 또는 Bayesian estimation 등) bootstrap 방법을 사용한다.

다음 단계는 가설적 측정모델이 수집된 데이터에 적합한지를 결정하는 것이다. 모델적합도를 나타내는 지수는 다양하고 그 평가기준도 전문가마다 조금씩 차이가 있다: χ^2^/degree of freedom, root mean square error of approximation, standardized root mean square residual, comparative fit index, normed fit index, goodness of fit index [7,8]. 국내 간호연구에서는 연구자가 사용한 모델적합지수의 평가기준과 연구자가 인용한 문헌에서 제시된 평가기준이 일치하지 않는 오류가 많이 발생한다.

측정모델 적합도가 만족되지 않으면, 연구자는 모델설정오류(model misspecification)의 가능성을 탐지하게 되며, 가설모델의 적합을 높이기 위해 주로 modification indices (MI)를 사용한다. Lee 등[9]은 당뇨병 관리에 대한 자기효능감 측정도구(Diabetes Management Self-Efficacy Scale, DMSES)의 구조타당도를 검증하기 위해 CFA를 사용하였다. 첫 번째 CFA 수행 결과, 모델적합지수가 기준보다 조금씩 부족하였다. 따라서 MI를 확인해 지수가 가장 높았던 문항 14와 16의 측정오차에 공분산을 설정하고 CFA를 다시 시행한 결과 모델이 유의하게 향상되었다. 이 후, DMSES 문항 16과 17, 그리고 문항 9와 10의 측정오차에 공분산을 차례로 설정하고 두 번의 CFA를 다시 실시하였다. 여기서 중요한 것은 여러 개의 모델수정을 한꺼번에 실시하는 것이 아니라, 하나의 모델수정이 실시될 때마다 기존 모델에 비해 유의하게 향상되었는지를 비교해야 한다. DMSES 연구에서는 기존모델과 수정모델의 Δχ^2^가 통계적으로 유의하게 감소했는지를 판단의 근거로 사용하였다. 그리고 연구자는 수정과 관련된 문항들 모두는 당뇨병 식이에 관한 것으로 문항 내용의 중복가능성을 제시하였다.

위의 예제와 같이 모델수정은 한 번에 하나씩 수행되어야 하며, 수정모형이 기존 모형보다 향상되었음을 근거에 입각해서 판단해야 한다. 또한 연구자는 수정된 현상에 대한 설명을 제시해야 한다. 하지만 국내 간호학술지에 게재된 도구개발 논문을 살펴보면, 거의 대부분의 연구에서 여러 개의 수정을 한번의 CFA를 통해 실시하고, 그 결과 사용된 다양한 모델적합도 지수 중 어떤 지수라도 조금 향상되기만 하면 이를 최종모델로 제시하고 있다. 또한 모델수정이 왜 발생했는지 또는 수정의 의미가 무엇인지에 대한 설명은 거의 찾아보기 힘들다.

모델이 전반적으로 적합하다면(overall model fit), 문항이 지정된 요인에 유의하게 적재되었는지(critical ratio value of >1.96)와 표준화된 요인적재 값이 .50 이상인지, 요인(잠재변수) 간의 관계가 너무 높지 않은지 살펴보아야 한다[10]. Lee 등[11]에 의한 Depression Anxiety Stress Scale (DASS)-12 구조타당도 검증을 사례로 살펴보자. DASS-12는 우울, 불안 및 스트레스 3개의 차원으로 구성된 도구다. CFA 결과 모든 문항이 지정된 요인에 유의하게 적재되었으며, 표준화 요인적재 값은 .655에서 .850로 높았다. 하지만 DASS-12의 우울과 불안 요인의 관계(φ=.887), 불안과 스트레스 요인의 관계(φ=.910), 그리고 스트레스와 우울 요인의 관계(φ=.910)가 모두 높게 나타났다. 이와 같이 요인들 간의 관계가 매우 높게 나타나면, 요인들을 합치거나 고차(higher-order) 모델의 가능성을 고려해야 한다. DASS-12의 경우 단일 요인을 가진 측정모델은 적합하지 않고 3개 요인으로 구성된 이차수준의 모델이 적합한 것으로 나타났으나, 3개 요인으로 구성된 기존의 일차수준의 CFA 모델과 비교해서 모델 향상이 통계적으로 유의하지 않았다. 따라서 최종적으로 DASS-12는 일차수준의 3개 요인으로 구성된 것으로 결론 내렸다.

간호학술지에 게재된 측정도구 논문을 살펴보면, CFA 결과에서 문항의 표준화된 요인적재 값이나 요인들 간의 관계에 문제가 있음에도 불구하고 연구자가 이에 대한 언급을 하지 않거나 또는 문제가 있음을 진술하고도 어떤 수정이나 해결 없이 타당도 검증을 끝내는 경우가 대부분이다. 2020년 간호학술지에 게재된 측정도구를 살펴보자[12]. 연구자는 CFA 결과 모형적합지수가 만족되었고, 요인적재 값이 .60 이상이므로 3개의 요인으로 구성된 구조타당도가 충족되었다고 하였다. 하지만 문항의 측정오차 6쌍에 대한 모델수정이 있었음에도 불구하고, 수정 과정 및 의미에 대한 언급이 전혀 없었다. 또한 잠재변수(요인)들 간의 높은 상관관계(.74.–.85)가 나타났는데, 다시 말해서 요인들이 독립되지 않았다는 것을 의미하는데도 불구하고 이에 대한 어떤 언급이나 추후 조치가 없었다.

때로는 문항의 표준화된 요인적재 값이나 요인들 간의 관계에 대한 이슈를 언급하기 위해 보조방법을 사용한다. 대표적인 것으로 경제학자인 Fornell과 Larcker [13]가 제안한 평균분산추출(average variance extracted, AVE)을 들 수 있다. 이들이 제시한 AVE 계산은 요인에 포함된 문항들의 표준화된 요인적재 값을 제곱한 합(∑요인적재값^2^)을 표준화된 요인적재 값의 제곱합과 측정오차의 합을 합한 값(∑요인적재값^2^+측정오차의 합)으로 나누는 것이다. Hair 등[10]은 ∑요인적재값^2^을 요인에 있는 문항의 수로 나누어 산출하는 간단한 방법을 제안하였다. 계산된 AVE가 .50보다 크면, 지정된 요인에서 문항들의 수렴타당성이 충족되었다고 하였다. 두 요인의 AVE 값들 모두가 요인 간에 공유된 분산(shared variance)보다 크면, (요인들 간의) 판별타당성이 만족된다고 볼 수 있다.

다시 위에서 언급한 연구[12]의 사례로 돌아가 보자. 이 연구에서 요인 1, 2, 3의 AVE를 각각 계산하면, .56, .65, .65가 된다. 따라서 각 요인의 문항들의 수렴타당성이 만족되었다고 할 수 있다. 하지만 요인들 간의 판별성에는 문제가 있다. 특히 요인 1과 2의 공유된 분산(요인들 간의 상관계수의 제곱)은 .72 (.85 × .85)로 요인 1과 2의 AVE .56이나 .65보다 높아서 요인 1은 2로부터(또는 요인 2는 1로부터) 분리하기 어렵다고 해석할 수 있다.

Lee 등[14]은 구조타당도에서 AVE 해석에 주의를 기울일 필요가 있다고 강조하였다. AVE를 이용한 문항의 수렴타당성 및 요인들 간의 판별타당성에 대한 해석은 모두 개발하고자 하는 도구의 내적구조(internal structure)를 확인하기 위한 보조방법이다. 그럼에도 불구하고 국내 일부 간호학자들은 개발하고자 하는 도구로 측정한 점수와 연구자가 가설에 의해 선택한 비교개념(comparator)을 측정하는 도구를 사용해서 수집한 점수가 어느 정도로 관련 있는가를 검증하는 외적 수렴타당도 및 판별타당도와 혼동해서 해석하는 경우가 있다고 하였다.

2021년 한 간호학술지에 게재된 Humanistic Practice Ability of Nursing (HPAN) 척도 개발 연구를 살펴보자[15]. 임상간호사 406명을 대상으로 자료를 수집해서 200명의 자료는 EFA 분석에, 나머지 116명의 자료는 CFA 분석에 사용하였다. 연구자는 HPAN 능력모델을 기틀로 사용하여 HPAN 척도를 개발하였다고 했다. 그렇다면 이미 가설로 제시한 측정모델이 있기 때문에 EFA 분석 없이 CFA를 수행할 수도 있었다. 더군다나 CFA에 사용할 표본크기가 116명으로 부족한 상황인데도 불구하고, 왜 직접 CFA를 수행하지 않았을까? 또한 도구개발을 위해 사용한 개념적 기틀에서 구성개념이 위계적 구조를 가지고 있다는 설명은 없었다(인용된 참고문헌은 중국어로 확인이 불가능하였음). 그럼에도 불구하고 가설로 제시된 측정모델은 왜 이차수준의 위계적 모델이었을까? 이차수준의 모델이 맞는다면, 5개의 요인으로 구성된 일차수준의 CFA로 분석한 이유는 무엇이었을까? 만약 연구자가 제시한 측정모형이 맞는다면(원문에 제시된 Figure 2), 문항들은 일차수준의 5개의 요인들과 이차수준의 요인 “humanistic practice ability of nursing”에 의해 설명되고, 각 문항은 지정된 일차요인에는 0이 아닌 값으로 적재되며(non-zero), 지정되지 않은 다른 4개의 일차수준의 요인에는 0으로 적재된다는 것을 의미한다. 또한 5개의 일차수준 요인들의 공변이는 전부 이차수준 요인 “humanistic practice ability of nursing”에 의해 설명된다는 것을 말하는 것이다. 이 연구의 측정모델에는 일차수준의 5개 요인들 간의 상관관계에 관한 것은 없음에도 불구하고, 연구자는 일차수준의 5개 요인의 AVE와 요인들 간의 공유된 분산 값을 사용하여 요인들의 판별타당성을 해석하였다. 다시 말해, 도구개발을 위해 사용한 개념적 기틀, 가설로 제시된 측정모델, 그리고 구조타당도를 검증한 방법의 연계성이 부족하다고 할 수 있다.

## 내적일관성

내적일관성(internal consistency)은 척도(하부척도)에 있는 문항들 모두가 자신들이 속한 그 잠재변수(요인)를 어느 정도로 측정하는지를 말한다[3]. 내적일관성 측정으로 가장 많이 사용되는 것은 Cronbach’s alpha이고, 도구개발 연구에서 측정속성으로 가장 많이 검증되는 것이기도 하다[14]. 하자만 측정도구가 다차원인 경우에 문항 전체에 대한 값만 제시하고 하부척도에 대한 α값을 제시하지 않는 경우들이 종종 있다. α값은 문항이 많을수록 증가하는 경향이 있다. 그러므로 측정도구가 다차원인 경우에는 전체문항 α값을 제시할 필요는 없으며, 각 하부척도 α를 제시해야 한다. 이와 관련해서 연구자들이 종종 범하는 오류는, 내적일관성 검증을 구조타당도 검증 전에 실시하는 것이다. 구조타당도 검증에서 측정도구의 차원(dimension)이 확정되어야 문항 전체에 대한 α값을 구할 것인지 아니면 하부척도 각각의 α값을 구할 것인지가 결정되기 때문이다. 최근에는 CFA를 통해 얻은 추정 값들을 이용한 omega index (ω)를 사용하기도 한다[16]. ω는 Cronbach’s alpha보다 조금 더 엄격한 방법으로, 보통 α값보다 약간 낮게 나타난다. 측정도구 문항반응이 이분형일 경우에 내적일관성 평가는 보통 Kuder-Richardson Formula 20을 사용한다[17]. 하지만 이분형 응답이 0과 1로 코딩되었다면, Cronbach’s alpha로 계산해도 상관없다[3].

위의 고전검사이론에 입각한 내적일관성 평가방법 이외에도 문항반응이론/라쉬 분석에서는 문항/피험자 분리신뢰도(item/person separation reliability or item/person reliability)와 문항/피험자 분리지수(person separation index)를 사용한다. 분리신뢰도는 0에서 1 사이의 값을 가지며 Cronbach’s alpha와 거의 유사한 값을 산출하고, 평가기준은 >.70이다. 분리지수는 0부터 무한대의 값을 가질 수 있는데, >1.50이면 수용할 만하다고 할 수 있다[18].

## 교차문화타당도/측정동일성

교차문화타당도/측정동일성(cross-cultural validity/measurement invariance)은 번역된 측정도구의 문항 또는 척도가 원래 측정도구에서의 문항/척도의 수행 정도를 얼마나 반영하는지에 관한 것이다[1]. 교차문화타당도/측정동일성을 검증하기 위해서는 연구 설계에 비교 집단이 포함되어야 한다. 예를 들어, 어떤 측정도구가 영어와 한국어 버전에서 척도/문항이 동일하게 작용하는지를 알아보기 위함이라면, 언어라는 비교 집단이 있어야 한다.

고전검사이론에서는 교차문화타당도/측정동일성 검증방법으로 다집단 확인적 요인분석(multi-group confirmatory factor analysis, MGCFA)을 많이 사용한다. 문항반응이론/라쉬 분석에서는 차별문항기능(differential item functioning, DIFF)이 사용된다[2].

만성질환자의 자가간호에 대한 자기효능감 측정도구(Self-care Self-efficacy Scale, SCSES)에 대한 교차문화타당도 검증 연구에 대해 살펴보자[19]. 미국, 중국(홍콩), 이탈리아 및 브라질의 만성질환자 957명으로부터 SCSES를 사용해서 자료를 수집하고, 교차문화타당도를 검증하기 위해 MGCFA를 사용해서 분석하였다. 그 결과 SCSES는 4개국 문화에 따라 측정의 동일성이 부분적으로 만족한 것으로 나타났다. 어떤 측정도구는 문화나 언어 이외에 성별, 나이 및 급·만성질환 등과 같이 대상자의 특성이나 조건에 따라 민감할 수 있다. 이 같은 도구의 속성을 파악하기 위해 측정동일성을 검증한다. Gomez 등[20]은 성별에 걸쳐서 DASS-21이 측정동일한지를 평가하기 위해서 687명의 성인(남자 227명, 여자 460명)을 대상으로 자료를 수집하고 MGCFA를 사용하여 분석하였다. 그 결과, DASS-21은 형태동일성(configural invariance) 및 요인계수동일성(metric invariance)을 만족하였다. 즉, 성별에 걸쳐 측정도구의 전반적인 요인구조가 같았고 문항-요인적재(item factor loading)의 강도가 동일하게 나타났다.

이번에는 문항반응이론의 견지에서 실시된 측정동일성 검증에 대해 살펴보자. Lindkvist 등[21]은 제1형 당뇨병을 진단받은 청소년을 대상으로 당뇨병 관리에 대한 자기효능감 도구(Self-efficacy in Diabetes Management, SEDM)를 검증하였다. SEDM은 총 10개의 문항으로 두 개의 하부척도로 구성되어 있다(요인 1: 문항 1-4번은 실제적 관리에 대한 자기효능감, 요인 2: 문항 5-10번은 정서적 자기효능감). 연구자는 SEDM의 문항이 성별집단에 관계없이 동일하게 기능하고 있는지를 확인하기 위해 라쉬 모델을 적용해서 DIFF를 실시하였다. 그 결과 요인 1에서는 성별에 따라 차별적으로 기능하는 문항이 없었으나, 요인 2의 문항 5, 9번은 차별적으로 기능하는 것으로 나타났다. 즉, 성별은 대상자들이 두 문항에 대해 어떻게 반응하는지에 영향을 준다는 것이다. 따라서 SEDS 점수계산은 성별에 따라 보정되어야 할 필요성이 있다는 것을 암시한다.

위와 같은 교차문화타당도/측정동일성에 관한 심리계량적 연구는 국내 간호학술지에서는 찾아보기 힘든 실정이다. 이에 대해 Lee 등[14]은 국내 간호학술지에 게재된 측정도구 연구는 고전적 검사이론에 입각한 구조타당도와 내적일관성 위주로 이루어지고 있다고 비평하였다. 따라서 앞으로 측정도구에 대한 심리계량적 연구를 할 때는, 다양한 측정 속성들에 대해 다양한 분석방법을 적용해서 평가해 볼 것을 권유한다.

## 요약

측정도구의 구조타당도는 내용타당도 이후 가장 먼저 수행되어야 할 측정 속성이다. 이를 위해 국내 간호학에서는 주로 CFA를 사용하는데, CFA로 얻을 수 없는 정보들을 제시하는 IRT/라쉬 분석도 같이 적용해 볼 것을 추천한다. 구조타당도 이외에 내적일관성 및 교차문화타당도/측정동일성 또한 측정도구의 내적구조를 확인하기 위한 것이다. 국내 간호학 측정도구 연구에서 교차문화타당도/측정동일성에 대한 검증은 거의 찾아볼 수 없는 상황이다. 따라서 앞으로는 이에 대한 평가도 시행되기를 바란다.
